# On the Virtues of James's Powder in the Apoplectic Diathesis

**Published:** 1818-06

**Authors:** 


### *On the Virtues of James's Powder in the Apoplectic Diathesis.* By J. Cheyne, M.D.

[ Dublin Hospital Reports. ]

Dr. Cheyne has been led to make several clinical experiments on the effects of the above-mentioned remedy in the apoplectic diathesis of aged persons, and his reports are favourable to its efficacy. He has also found James's

Powder of remarkable utility in certain instances of determination of blood to the head, occurring in the early periods of life, and threatening to end in effusion. Lastly, in two cases of general plethora, in which, however, the head was more affected than any other part, this medicine was exhibited by Dr. Cheyne with perfect success. The following is an Outline of one of these Cases.

A lady, about 28 years of age, of sanguine temperament and full habit, had been affected, for nearly seven years, with a distressing fulness in her head. During the first three years she bare three still-born children. She then went to London, and consulted an eminent accoucheur, who ordered her to live low, abstain from fermented liquors, and be bled whenever her head was severely affected. "In the two following years she had two children born healthy and strong." The next two years, though on the same plan of treatment, were not so well passed, and she again had a still-born child.

She could now hardly keep her eyes open, in consequence of the heat and weight in her head. She had continued vertigo; indistinct vision; impaired memory, with stupor and confusion; had a sense of suffocation and fulness about her throat, so that she feared to go to sleep, lest she should never awake. But what distressed her more than all, was a peculiar sensation at her heart, "as if there was no pulse in it for nearly a minute—as if it had not room to beat." Her skin was dry and hot; pulse full, and rather quick, with swelling, but not œdema of the hands and feet.

She began a course of James's Powder, by taking two grains the first night, and half a grain additional every succeeding night, till the dose amounted to twenty grains, which she continued to take for five weeks, when she found herself so well that she discontinued the medicine. Meantime she got clear of all her unpleasant symptoms. The medicine did not produce any perceptible effect. In removing the dry and burning heat of the skin it did not sensibly produce perspiration.

Dr. Cheyne generally directs the patient to begin with two grains at bed-time, increasing the dose by half a grain every night until some sensible effect be produced on the stomach, bowels, or skin. When gastric irritability supervenes, the dose is to be lessened the succeeding night. By combining a few grains of rhubarb with this medicine it may be borne in a larger dose than when exhibited alone. If the skin be softened, or the bowels affected, the dose should not be further increased, but it must be repeated every night for a considerable length of time. In one or two instances it had a soporific effect. It has not been productive of any injurious effects upon the appetite or digestion; and no rules of diet, except the avoiding of acids, have been given beyond what the case otherwise required. It did not appear that the habit was more disposed to catarrhal or rheumatic complaints while under the influence of James's Powder, than at any other time.

This medicine is not recommended to supersede the usual remedies—bleeding, purging, and a strict antiphlogistic regimen, but merely to form a part of the prophylactic treatment when the threatened fit of apoplexy is over.

It would not be difficult to explain the good effects of antimonials in relieving *local* congestions or determinations, on the principle of their deriving to the *periphery* of the body, promoting intestinal secretions, and controlling increased vascular actions: but this we need not dwell on at present.

Dr. C. has not used any other preparations of antimony than James's Powder, as they are not so uniform in their effects, though he thinks they might be substituted in œconomic prescriptions. The same remedy is equally applicable to epilepsy connected with the apoplectic diathesis, and perhaps to other species of epilepsy also.

This paper, like all the others of Dr. Cheyne in the same volume, is characterized by accurate observation and clear judgment: and we may here state our opinion, that the DUBLIN HOSPITAL REPORTS, as a whole, may competite with any volume of a similar description in the English language. We wish the work every success.

